# Smart Hydrogel Formed by Alginate-*g*-Poly(*N*-isopropylacrylamide) and Chitosan through Polyelectrolyte Complexation and Its Controlled Release Properties

**DOI:** 10.3390/gels8070441

**Published:** 2022-07-14

**Authors:** Min Liu, Jingling Zhu, Xia Song, Yuting Wen, Jun Li

**Affiliations:** 1 Department of Biomedical Engineering, National University of Singapore, 15 Kent Ridge Crescent, Singapore 119276, Singapore; a0113478@u.nus.edu (M.L.); erizhuj@nus.edu.sg (J.Z.); a0045788@u.nus.edu (X.S.); bieweny@nus.edu.sg (Y.W.); 2 NUS Graduate School for Integrative Sciences & Engineering (NGS), National University of Singapore, 28 Medical Drive, Singapore 117456, Singapore

**Keywords:** polysaccharides, anionic polymer, cationic polymer, thermosensitive, pH-sensitive, drug delivery

## Abstract

Smart hydrogels that can respond to external stimuli such as temperature and pH have attracted tremendous interest for biological and biomedical applications. In this work, we synthesized two alginate-*graft*-poly(*N*-isopropylacrylamide) (Alg-*g*-PNIPAAm) copolymers and aimed to prepare smart hydrogels through formation of polyelectrolyte complex (PEC) between the negatively charged Alg-*g*-PNIPAAm copolymers and the positively charged chitosan (Cts) in aqueous solutions. The hydrogels were expected to be able to respond to both temperature and pH changes due to the nature of Alg-*g*-PNIPAAm and chitosan. The hydrogel formation was determined by a test tube inverting method and confirmed by the rheological measurements. The rheological measurements showed that the PEC hydrogels formed at room temperature could be further enhanced by increasing temperature over the lower critical solution temperature (LCST) of PNIPAAm, because PNIPAAm would change from hydrophilic to hydrophobic upon increasing temperature over its LCST, and the hydrophobic interaction between the PNIPAAm segments may act as additional physical crosslinking. The controlled release properties of the hydrogels were studied by using the organic dye rhodamine B (RB) as a model drug at different pH. The PEC hydrogels could sustain the RB release more efficiently at neutral pH. Both low pH and high pH weakened the PEC hydrogels, and resulted in less sustained release profiles. The release kinetics data were found to fit well to the Krosmyer–Peppas power law model. The analysis of the release kinetic parameters obtained by the modelling indicates that the release of RB from the PEC hydrogels followed mechanisms combining diffusion and dissolution of the hydrogels, but the release was mainly governed by diffusion with less dissolution at pH 7.4 when the PEC hydrogels were stronger and stabler than those at pH 5.0 and 10.0. Therefore, the PEC hydrogels are a kind of smart hydrogels holding great potential for drug delivery applications.

## 1. Introduction

Smart hydrogels that can sense and respond to external stimuli, such as temperature, pH, light, and electric fields, have attracted tremendous interest because of their great potential for biological and biomedical applications [1,2,3,4,5,6,7,8,9]. Their properties, such as mechanical stability and release kinetics, can be tailored for desired applications [3,10,11,12,13]. Based on external stimuli type, they can be classified as thermo-sensitive, pH sensitive, electro-sensitive, and others [14]. Thermo-sensitive and pH sensitive hydrogels are the most extensively studied polymer systems. The presence of thermo-sensitive hydrophilic–hydrophobic switchable groups is the characteristic of thermo-sensitive hydrogels. For example, the reversible phase transition of poly(*N*-isopropylacrylamide) (PNIPAAm) in response to temperature changes makes its copolymers form thermoresponsive hydrogels [15,16,17,18,19]. PNIPAAm is an extensively studied thermoresponsive polymer with a lower critical solution temperature (LCST) of 32 °C [15,19,20,21]. It is hydrophilic at a temperature below its LCST, and becomes hydrophobic at a temperature above the LCST [20,22]. The LCST of PNIPAAm could be altered by its copolymers with hydrophilic or hydrophobic polymers [20,23,24]. Copolymerization with a hydrophobic polymer would decrease the LCST, while copolymerization with a hydrophilic polymer would increase the LCST [25,26].

Alginate (Alg) has been used to copolymerize with PNIPAAm and the copolymers have been reported to form thermoresponsive micelles and hydrogels for drug delivery and other applications [27,28,29,30,31,32,33]. The naturally occurring polysaccharide alginic acid is a liner copolymer of 1–4 linked α-L-guluronic acid (G) and β-D-mannuronic acid (M), which is biocompatible, biodegradable, non-toxic, chelatable, and suitable for chemical modification [30]. The carboxyl groups of alginic acid are pH sensitive [34]. The pKa values of the M and G carboxyl groups are 3.65 and 3.38, respectively [35]. The hydrophilic and hydrophobic units along a molecular chain can be altered by the protonation and deprotonation of the carboxyl groups [35]. Deprotonation at higher pH than the pKa will lead to ionization which in turn, increases the electrostatic repulsion and hydrophilicity [14,36,37]. 

Sodium alginate has been reported to form polyelectrolyte complexes (PECs) with chitosan (Cts) [38,39,40,41,42]. Chitosan is a naturally derived polysaccharide copolymer of N-acetyl-D-glucosamine and D-glucosamine. The pKa value of the D-glucosamine residue is about 6.2–7.0 [40,42]. As a result, chitosan is soluble in acidic aqueous solutions [40,42,43,44]. Based on the pKa values of chitosan and alginic acid, a strong ionic interaction between the positively charged amine groups of chitosan and the negatively charged carboxylate groups of alginate should be formed in the pH range from 4 to 6 [43]. The strong electrostatic interaction between a cationic polymer and an anionic polymer has been also applied for condensing and delivering nucleic acids, which are negatively charged polymers, using cationic polymers as gene carriers for gene therapy through formation of PEC-based nanogels and nanoparticles [45,46,47,48,49,50]. 

**Figure 1:**
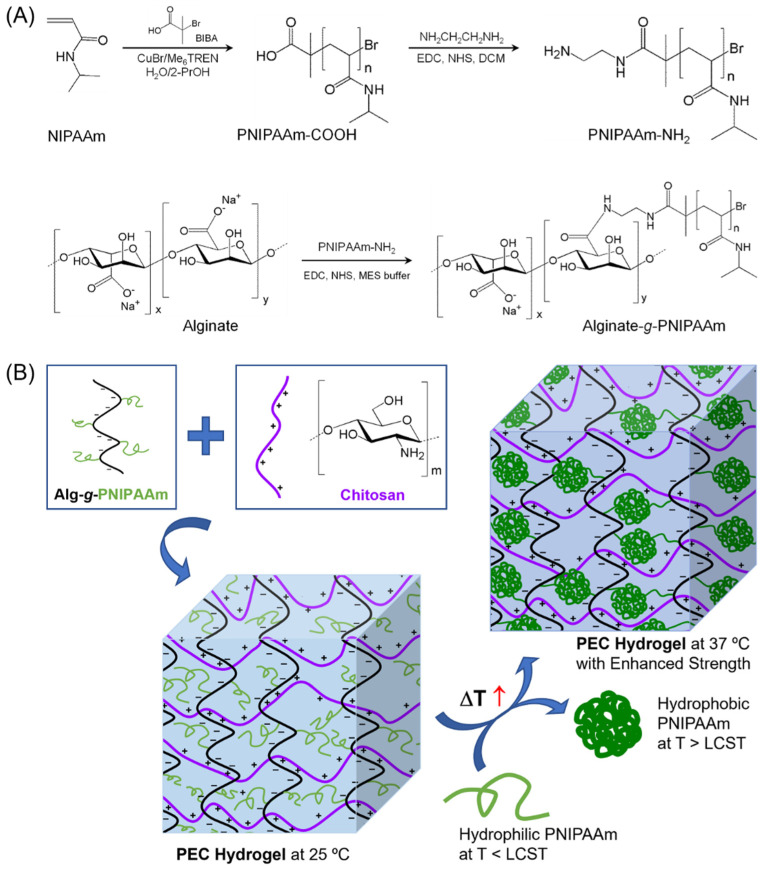
(**A**) Synthesis scheme for alginate-g-poly(N-isopropylacrylamide) (Alg-*g*-PNIPAAm) copolymers. (**B**) Formation of polyelectrolyte complex (PEC) hydrogels between Alg-*g*-PNIPAAm and chitosan, and the transition of PNIPAAm segments from hydrophilic at 25 °C to hydrophobic at 37 °C, which enhances the PEC hydrogels due to additional physical crosslinking. Abbreviations: NIPAAm—*N*-isopropylacrylamide; BIBA—α-bromoisobutyric acid; Me_6_TREN—tris [2-(dimethylamino)ethyl]amine; PrOH—isopropanol; EDC—1-ethyl-3-(dimethylaminopropyl)carbodiimide; NHS—*N*-hydroxysuccinimide; DCM—dichloromethane; MES—2-(*N*-morpholino)-ethanesulfonic acid; LCST—lower critical solution temperature.

Previously we synthesized Alg-*g*-PNIPAAm thermosensitive copolymers (Figure 1A), and found that the copolymers form injectable thermoresponsive hydrogels, which could encapsulate the anti-cancer drug doxorubicin [28]. Interestingly, the drug-loaded hydrogels could form doxorubicin-encapsulated micelles upon dilution in aqueous medium at the body temperature 37 °C, and efficiently deliver the drug into cancer cells through the micelles. The Alg-*g*-PNIPAAm thermosensitive copolymers were also used as a smart thermoresponsive adsorption system for efficiently removing copper ion from wastewater [29].

Herein, we aimed to prepare smart hydrogels with dual responsiveness to both temperature and pH through formation of PEC between the Alg-*g*-PNIPAAm copolymers and chitosan. We hypothesize that the negatively charged Alg-*g*-PNIPAAm copolymers can form PEC with the positively charged chitosan in aqueous solutions, and further form hydrogels that are composed of a network structure primarily induced by the PECs between the alginate segments of Alg-*g*-PNIPAAm and the positively charged chitosan polymer segments. In addition, the Cts/Alg-*g*-PNIPAAm PEC hydrogels formed at room temperature may be further enhanced by increasing the temperature over the LCST of PNIPAAm, because PNIPAAm will change from hydrophilic to hydrophobic upon temperature increase over its LCST, and the hydrophobic interaction between the PNIPAAm segments may play a role as additional physical crosslinking (Figure 1B). 

In this report, PEC hydrogels were successfully prepared by mixing the Alg-*g*-PNIPAAm copolymers and chitosan in aqueous solutions. The rheological properties and the strength and stability of the Cts/Alg-*g*-PNIPAAm PEC hydrogels were studied. The rheological tests revealed that the PEC hydrogels were much stronger than the counterpart non-PEC hydrogels formed by Alg-*g*-PNIPAAm alone, and that the PEC hydrogels were further enhanced by the hydrophobic interactions of the PNIPAAm segments upon increasing the temperature from 25 to 37 °C. It is well-known that a hydrogel has great potential for drug delivery applications. Herein, we also studied the controlled release properties of the Cts/Alg-*g*-PNIPAAm PEC hydrogels using the organic dye rhodamine B (RB) as a model drug. Since both alginate and chitosan are pH-sensitive polymers, the drug release experiments were carried at different pH to study the effect of pH on the release kinetics.

## 2. Experimental Section

### 2.1. Materials

Sodium alginate (M/G = 1.56, M_n_ = 120k–190k Da) was purchased from Sigma-Aldrich. Alginate-*g*-PNIPAAm copolymers were synthesized as we reported previously [28]. Chitosan (M_n_ = 50–190 kDa), rhodamine B, and 2-(*N*-morpholino)-ethanesulfonic acid (MES) were obtained from Sigma-Aldrich.

### 2.2. Synthesis of Alginate-g-PNIPAAm

The detailed synthesis procedures and characterization methods for the copolymers can be found in the electronic Supplementary Materials. Two alginate-*g*-PNIPAAm copolymers with different PNIPAAm lengths, degrees of substitution (DS), and PMIPAAm contents were synthesized and used in this study (Table 1).

### 2.3. Hydrogel Formation

The PEC hydrogels formed by alginate-*g*-PNIPAAm and chitosan were prepared by mixing an aqueous solution of alginate-*g*-PNIPAAm with acetic acid solution of chitosan at room temperature. The weight ratio of alginate segment to chitosan was set to be 17:13.8 which converts to a 1:1 molar ratio carboxylate group of alginate to amine group of chitosan. The total content of polymers (alginate-*g*-PNIPAAm and chitosan) was fixed to be 7.4 wt%. The hydrogels formed by alginate-*g*-PNIPAAm alone were prepared by dissolving alginate-*g*-PNIPAAm in water, followed by adding acetic acid solution equivalent to the amount used for dissolving chitosan in the preparation of the chitosan/alginate-*g*-PNIPAAm PEC hydrogels. The final copolymer contents of the hydrogels were 7.4 wt%. The hydrogel formation was determined by a test tube inverting method. Each sample for hydrogel preparation was carried out in a test tube. The same was considered to have formed hydrogel if it could not flow even when the sample tube was inverted by 180°.

### 2.4. Rheological Studies

The dynamic rheological measurements were carried out using a HAAKE™ MARS III Rotational Rheometer with parallel plate geometry (35 mm diameter) at a gap of 1.0 mm. Samples were prepared as described in the previous section. The samples were carefully loaded onto the measuring geometry and oil was added around the measuring geometry to minimize the water evaporation during the experiments. The changes in elastic modulus (G′) and viscous modulus (G″) were measured at a fixed frequency of 1.0 Hz and a constant stress of 1.0 Pa with heating from 25 to 37 °C at 0.05 °C/s. Oscillatory stress sweeps were carried out by applying an increasing shear stress logarithmically from 0.1 Pa at a fixed frequency of 1.0 Hz at 37 °C, until the hydrogels were destroyed at a G′/G″ crossover, and a 100% deformation was reached. The yield stress (τ) was defined as the applied shear stress at the G′/G″ crossover [51].

### 2.5. In Vitro Release of Rhodamine B from Hydrogels

Rhodamine B (RB) was dissolved in deionized water at 2 mg/mL. Alginate-*g*-PNIPAAm was dissolved in the RB solution. The PEC hydrogels formed by alginate-*g*-PNIPAAm and chitosan were prepared by mixing the aqueous solution of alginate-*g*-PNIPAAm with an acetic acid solution of chitosan at room temperature. The weight ratio of alginate segment to chitosan was set to be 17:13.8, which converts to a 1:1 molar ratio of carboxylate group of alginate to amine group of chitosan. The total content of polymers (alginate-*g*-PNIPAAm and chitosan) was 7.4 wt% and the content of RB was 0.1 wt%. The hydrogels formed by alginate-*g*-PNIPAAm alone were prepared by dissolving alginate-*g*-PNIPAAm in the RB solution, followed by adding an acetic acid solution equivalent to the amount used for dissolving chitosan in the preparation of the chitosan/alginate-*g*-PNIPAAm PEC hydrogels. The final copolymer contents of the hydrogels were 7.4 wt% and the content of RB was 0.1 wt%.

The pre-weighted hydrogels (500 mg) were loaded into a 1 mL syringe. The syringe was incubated at 37 °C for 15 min. Then, the hydrogel was injected into a tea bag and the tea bag was immersed into 30 mL release medium in a centrifuge tube. The sample tube was shaken at 100 rpm at 37 °C in the dark. At predetermined time intervals, 10 mL of release medium was replaced by fresh release medium. The concentration of RB in the release medium was calculated by measuring the absorbance of RB at 555 nm using a microplate reader (Infinite M200 PRO, TECAN). RB solutions of various concentrations in PBS buffer were prepared and absorbance at 555 nm was measured to produce a standard curve. From the standard curve the following equation was obtained, where C_RB1_ is the concentration of RB and A_1_ is the absorbance value. The calibration curve under the concentration range from 0.00039 to 0.025 mg/mL is linear with a correlation coefficient of R^2^ = 0.9999.

C_RB1_ = 20.846A_1_ − 0.5753

The cumulative drug release was calculated from the following equation:

Cumulative release (%) = M_t_/M_0_ × 100

here, M_t_ is the total amount of RB released from the hydrogels at time t, and M_0_ is the amount of RB loaded into the hydrogel.

After 100% release of RB from the hydrogels, the tea bags were removed from the release media. The tea bags were weighed after freeze-drying, and the residual polymers of the hydrogels were calculated. The weight percentage of residual polymers in the tea bags after release was calculated from the following equation:

Residual polymer (%) = M_f,p_/M_0,p_ × 100

here, M_f,p_ is the amount of polymer remained in the tea bag after release, and M_0,p_ is the initial amount of polymer in the hydrogel.

### 2.6. Release of Rhodamine B from Hydrogels at Different pH

The release medium was changed from PBS (pH 7.4) to acetic acid/sodium acetate solution (acetic buffer, pH 5.0) and sodium tetraborate/sodium hydroxide solution (borate buffer, pH 10.0). The linear correlation of RB concentration C_RB2_ and corresponding absorbance A_2_ in the acidic solution is below with R^2^ = 0.9998:

C_RB2_ = 20.728A_2_ − 0.5339

The linear correlation of RB concentration C_RB3_ and corresponding absorbance A_3_ in the basic solution is below with R^2^ = 0.9999:

C_RB3_ = 21.877A_3_ − 0.656

### 2.7. Modelling of Release Kinetics and Mechanism

The release kinetics and mechanism of RB released from the hydrogels was simulated using a mathematical model well reported in the literature [52], which is the Krosmyer–Peppas model that is expressed by the following equation:

*M_t_*/*M*_0_ = *Kt^n^*

where *M_t_* is the total amount of RB released from the hydrogel at time *t*, *M*_0_ is the amount of RB loaded into the hydrogel, *M_t_*/*M*_0_ is the fraction of RB released from the hydrogel at time *t*, *K* is the release rate constant, and *n* is the release exponent.

The Krosmyer–Peppas model is an advanced model that includes Fickian diffusion mechanism (*n* = 0.5), anomalous non-Fickian transport mechanism (0.5 < *n* < 1.0), non-Fickian Case II relaxational transport (zero-order release, *n* = 1.0), and super case II transport mechanism (*n* > 1.0).

## 3. Results and Discussions

### 3.1. Synthesis and Characterization of Alginate-g-PNIPAAm

Two alginate-g-PNIPAAm copolymers with different PNIPAAm lengths, degrees of substitution (DS), and PNIPAAm contents were synthesized using the method we reported previously [28]. The synthesis scheme is shown in Figure 1A. The molecular characteristics of the copolymers measured from their ^1^H NMR spectra (Figure S1) are listed in Table 1. The characteristic peaks of PNIPAAm are located at 3.82 ppm (-NH-CH) [53], 1.07 ppm (-CH_3_) [31], 1.94 ppm (-CH-), and 1.50 ppm (-CH_2_-), while those of NIPAAm monomer between 5.0 and 6.0 ppm disappeared. PNIPAAm with amino end was synthesized by introducing excessive ethylenediamine (EDA) to the end of PNIPAAm-COOH via carbodiimide chemistry. The extra EDA was removed by precipitation in diethyl ether. The clearance of EDA was confirmed by thin layer chromatography with acetone as solvent. The amino end group was quantified by the TNBS assay [54]. The grafting of PNIPAAm-NH_2_ to alginate was confirmed by ^1^H NMR spectroscopy (Figure S1). Three protons of the alginate ring (i.e., H2, H3, and H4) are overlapped with characteristic signals of PNIPAAm-NH2 at around 3.8 ppm [27,32,33].

### 3.2. PEC Hydrogel Formation

**Figure 2:**
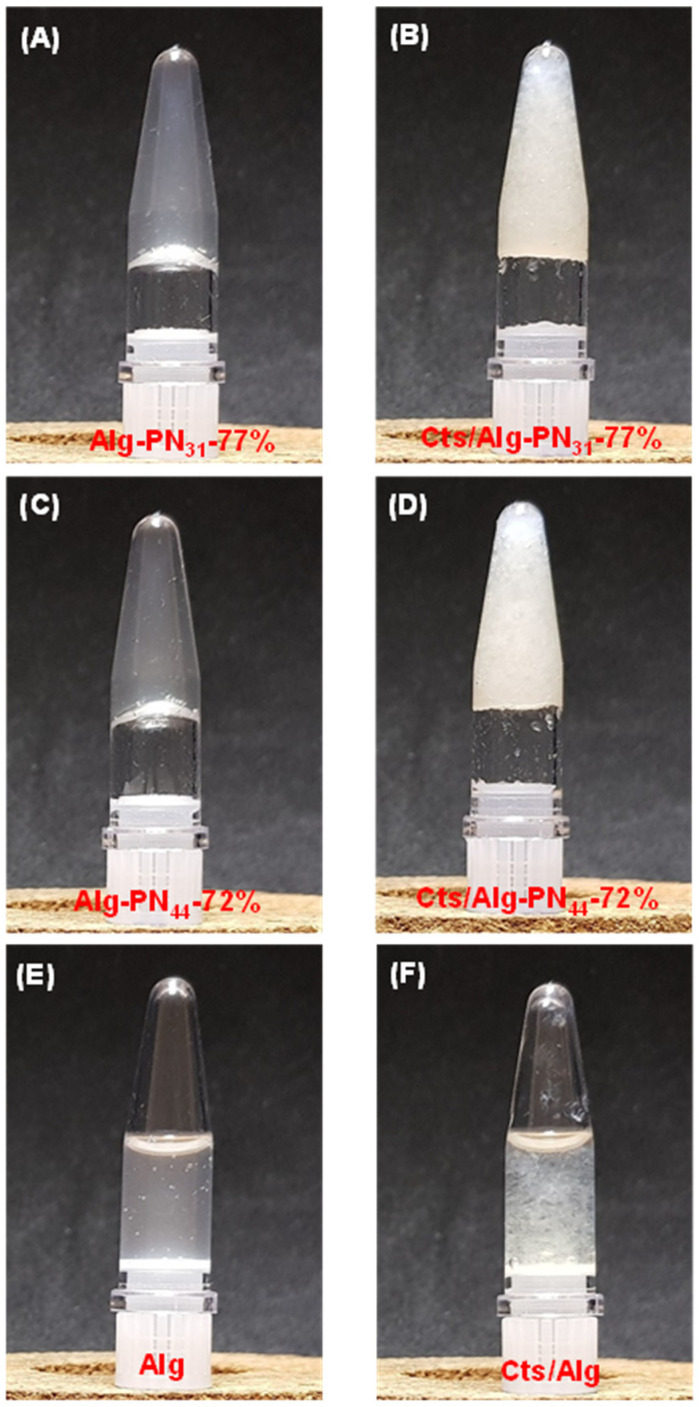
Photographs of copolymer hydrogels and PEC hydrogels formed at room temperature: (**A**) Alg-PN_31_-77% copolymer hydrogel; (**B**) Cts/Alg-PN_31_-77% PEC hydrogel; (**C**) Alg-PN_44_-72% copolymer hydrogel; (**D**) Cts/Alg-PN_44_-72% PEC hydrogel; (**E**) Alg sol; and (**F**) Cts/Alg sol.

The formulations of the samples used for the PEC hydrogel formation are listed in Table 2. The sol-gel transition was determined by a test tube inverting method. At room temperature (25 °C), the Alg-PN_31_-77% and Alg-PN_44_-72% copolymers formed solutions at 7.4 wt% in PBS buffer (pH = 7.4), which could flow easily in sample test tube, as we reported previously [28]. However, weak hydrogels were formed by Alg-PN_31_-77% and Alg-PN_44_-72% at 7.4 wt% when the copolymers were dissolved in acetic acid solutions at room temperature (Figure 2A,C). It is thought that the carboxylate groups of alginate in the Alg-*g*-PNIPAAm copolymers were protonated and became partially hydrophobic, acting as physical crosslinking. This is consistent with the previous reports which claim that alginate could form hydrophobic core [35] and may aggregate to form a fine gel at pH 4–5 [6]. With the addition of chitosan, both Alg-PN_31_-77% and Alg-PN_44_-72% copolymers formed stronger PEC hydrogels with chitosan at room temperature (Figure 2B,D). The sample Alg containing the same alginate content as that in Cts/Alg-PN_44_-72% PEC hydrogel, and the sample Cts/Alg containing the same alginate content and chitosan content as those in Cts/Alg-PN_44_-72% PEC hydrogel, could not form hydrogels at room temperature (Figure 2E,F).

### 3.3. Rheological Study

**Figure 3:**
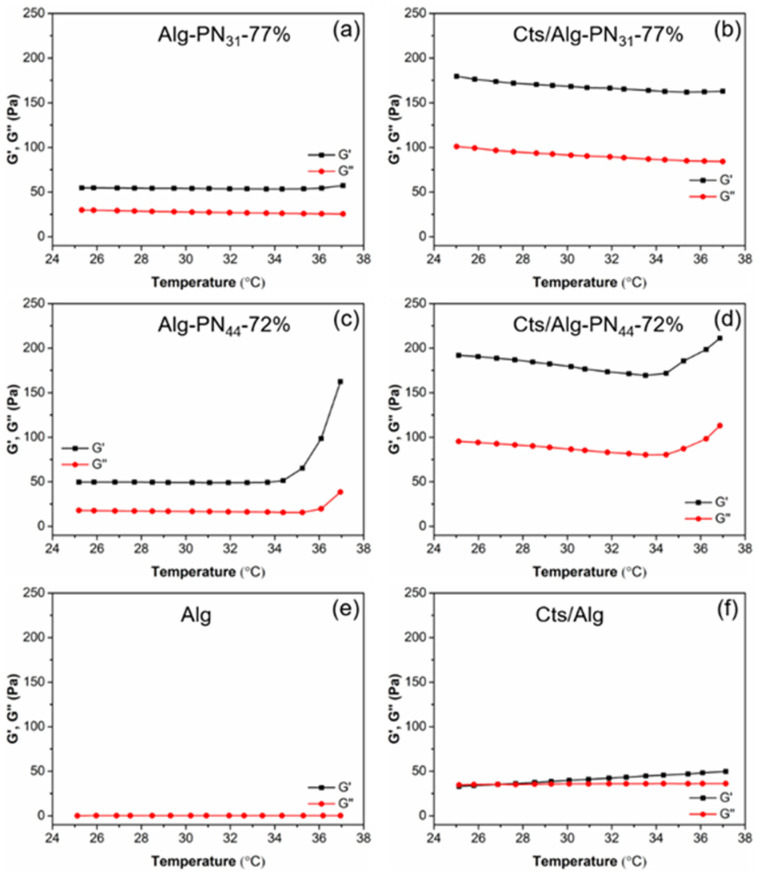
Elastic modulus (G′) and viscous modulus (G″) for Alg-PN_31_-77% (**a**), Cts/Alg-PN_31_-77% (**b**), Alg-PN_44_-72% (**c**), Cts/Alg-PN_44_-72% (**d**), Alg (**e**), and Cts/Alg (**f**) measured with a constant frequency (1.0 Hz) and fixed stress (1.0 Pa) at the plate temperature increasing from 25 to 37 °C at a rate of 0.05 °C/s.

**Figure 4:**
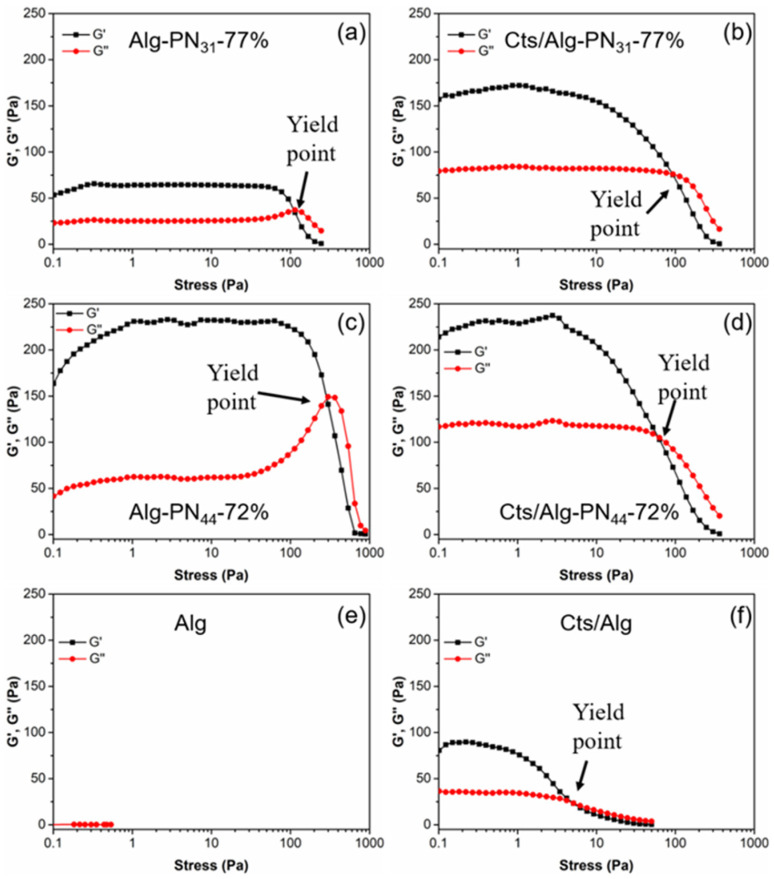
Oscillatory stress sweep measurements of hydrogel samples Alg-PN_31_-77% (**a**), Cts/Alg-PN_31_-77% (**b**), Alg-PN_44_-72% (**c**), Cts/Alg-PN_44_-72% (**d**), Alg (**e**), and Cts/Alg (**f**). The measurements were performed with a constant frequency (1.0 Hz) at 37 °C.

The rheological study of the samples in Table 2 were carried out and the results are shown in Figure 3 and Figure 4. Generally, with the addition of chitosan into Alg-*g*-PNIPAAm copolymers, the PEC hydrogels were formed with much higher strength compared to the corresponding copolymer hydrogels. Moreover, the strength of the hydrogels Alg-PN_44_-72% and Cts/Alg-PN_44_-72% significantly increased with the increase of temperature up to 37 °C. Moreover, the PEC hydrogels formed by the Alg-*g*-PNIPAAm copolymers were more resistant to shear stress than the sample Cts/Alg.

The elastic modulus (G’) of the PEC Cts/Alg-PN_31_-77% hydrogel was about 3.5 times larger than that of the Alg-PN_31_-77% copolymer hydrogel at temperature ranging from 25 to 37 °C (Figure 3a,b). There was no clear increase in gel strength with the increase in temperature. When comparing the PEC Cts/Alg-PN_44_-72% hydrogel with the copolymer Alg-PN_44_-72% hydrogel, it is found that the G’ of the PEC Cts/Alg-PN_44_-72% hydrogel was nearly four times larger than that of the copolymer Alg-PN_44_-72% hydrogel at temperature ranging from 25 to 34 °C. It is noteworthy that the G’ for both Alg-PN_44_-72% and Cts/Alg-PN_44_-72% significantly increased when the temperature reached over 34 °C (Figure 3c,d). The sample Cts/Alg in Figure 2F formed PEC but could not gel at room temperature. However, it formed a very weak hydrogel at temperature 27 °C or above, with G’ values of 40 to 50 Pa, which are slightly higher than its G” (Figure 3f).

From the results shown in Figure 3a–d, we can conclude that 1) PEC hydrogels form by Alg-*g*-PNIPAAm and chitosan are much stronger that the hydrogels formed by Alg-*g*-PNIPAAm alone, apparently because of the strong electrostatic interaction between the negatively charged alginate segments and the positively charged chitosan chains; and 2) the hydrophobic interaction between the PNIPAAm segments at temperatures above its LCST can act as additional physical crosslinking, which largely strengthen the hydrogels (both Alg-PN_44_-72% and Cts/Alg-PN_44_-72% hydrogels), making the hydrogels thermosensitive, or “smart” in responding to temperature changes around the LCST of PNIPAAm. The hydrogels formed by Alg-PN_44_-72% (both Alg-PN_44_-72% and Cts/Alg-PN_44_-72% hydrogels) showed clear thermosensitive property but those by Alg-PN_31_-77% did not, indicating that longer PNIPAAm graft chains are necessary for an Alg-*g*-PNIPAAm copolymer to form thermosensitive hydrogels under the conditions explored in this study. Similar observations were also reported by us [10,28] and others [15,17,19,20,22,30,55].

The yield stress σ_y_ is the shear stress required to destroy the hydrogel network, which happens at the G′/G″ crossover when the solid-like hydrogel changes into a liquid-like one [56]. The yield stress σ_y_ was 5.1 pa for Cts/Alg (Figure 4f). It was the smallest value among the hydrogels tested in this study. From this, we understand that the polyelectrolyte complexation formed between the carboxylate groups of alginate and the amine groups of chitosan could be destroyed easily under the shear stress. The hydrogels in Figure 4a–d containing thermoresponsive PNIPAAm chains had much higher yield points due to the hydrophobic interactions of PNIPAAm at 37 °C. The longer PNIPAAm chains of Alg-PN_44_-72% gave higher yield point than that of Alg-PN_31_-77% (Figure 4a,c).

When comparing Alg-PN_31_-77% and Alg-PN_44_-72% hydrogels (Figure 4a,c) with Alg alone (Figure 4e), PNIPAAm grafting to Alg contributed much to the high yield stress. In the PEC hydrogels the content of Alg-*g*-PNIPAAm polymer is less than those in the Alg-*g*-PNIPAAm hydrogels, but yield stress values were similar to those of the Alg-*g*-PNIPAAm hydrogels, which can be considered an effect of the combination of PEC and hydrophobic interaction of PNIPAAm segments.

### 3.4. In Vitro Release of Rhodamine B from Hydrogels

**Figure 5:**
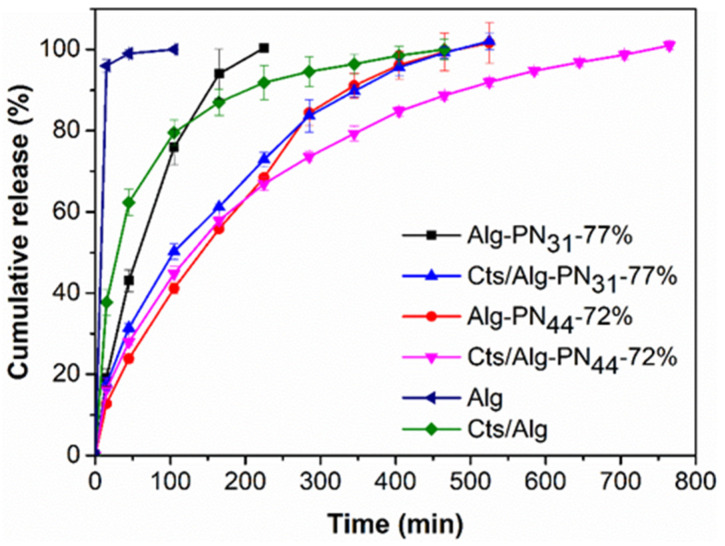
Cumulative release of rhodamine B from the hydrogel samples Alg-PN_31_-77%, Cts/Alg-PN_31_-77%, Alg-PN_44_-72%, Cts/Alg-PN_44_-72%, Alg, and Cts/Alg in PBS buffer (pH 7.4) at 37 °C.

The pre-weighted hydrogel with rhodamine B encapsulated was loaded into a 1 mL syringe and incubated at 37 °C for 15 min. Then, it was injected into a tea bag and the tea bag was immersed into 30 mL of PBS release buffer (pH 7.4) in a covered test tube, which was incubated at 37 °C in a water bath shaking at 100 rpm. The rhodamine B (RB) release profiles from the hydrogels are shown in Figure 5. The weight percentages of polymers left after 100% release of RB are listed in Table 3.

Generally, at pH 7.4 among the six hydrogels or polymers tested for RB release, they provided more sustained release in the order Cts/Alg-PN_44_-72% > Alg-PN_44_-72% > Cts/Alg-PN_31_-77% >> Cts/Alg > Alg-PN_31_-77% > Alg. Longer PNIPAAm chain in the Alg-*g*-PNIPAAm copolymer and the formation of PEC could make the hydrogels stronger and stabler, thus reducing the release rate of RB from the hydrogels. So, Alg-PN_44_-72% exhibited more sustained release than Alg-PN_31_-77%, and Cts/Alg-PN_44_-72% and Cts/Alg-PN_31_-77% more than Alg-PN_44_-72% and Alg-PN_31_-77%, respectively.

The hydrogels formed by alginate-*g*-PNIPAAm alone eroded completely after the complete release of RB, whereas 40–70% of the PEC hydrogels remained after the complete release of RB. As a result, the release rates of RB from those non-PEC hydrogels were faster than those corresponding PEC hydrogels. It is thought that the RB release rate depended on the diffusion rate as well as the hydrogel eroding rate. The formation of PEC slowed down the eroding of the hydrogels and resulted in a more sustained release of RB. In a recent report, Cts/Alg PEC hydrogels were additionally crosslinked with divalent cations such as Ca^2+^ and Zn^2+^ for enhancing the drug release properties [57]. our PNIPAAm grafting to Alg approach has provided an alternative to make Cts/Alg PEC hydrogels suitable for sustained drug release.

### 3.5. Effect of pH on Release Profiles

**Figure 6:**
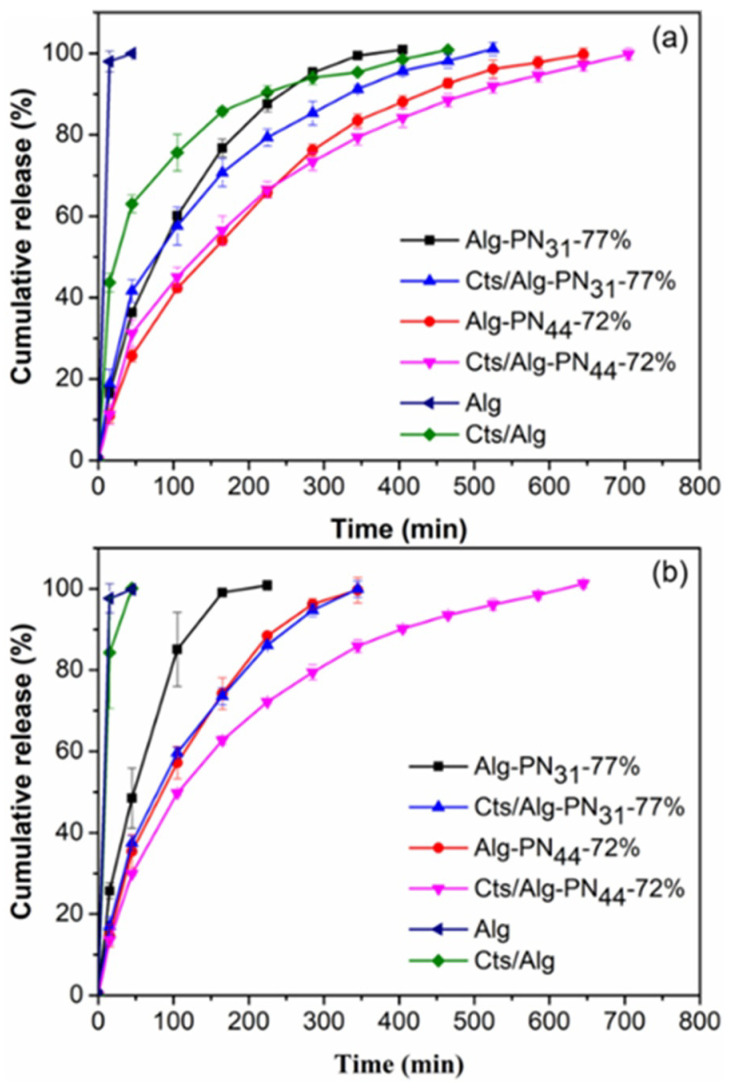
Cumulative release of rhodamine B from the hydrogel samples Alg-PN_31_-77%, Cts/Alg-PN_31_-77%, Alg-PN_44_-72%, Cts/Alg-PN_44_-72%, Alg, and Cts/Alg in (**a**) acetic buffer (pH 5.0), and (**b**) borate buffer (pH 10.0) at 37 °C.

**Figure 7:**
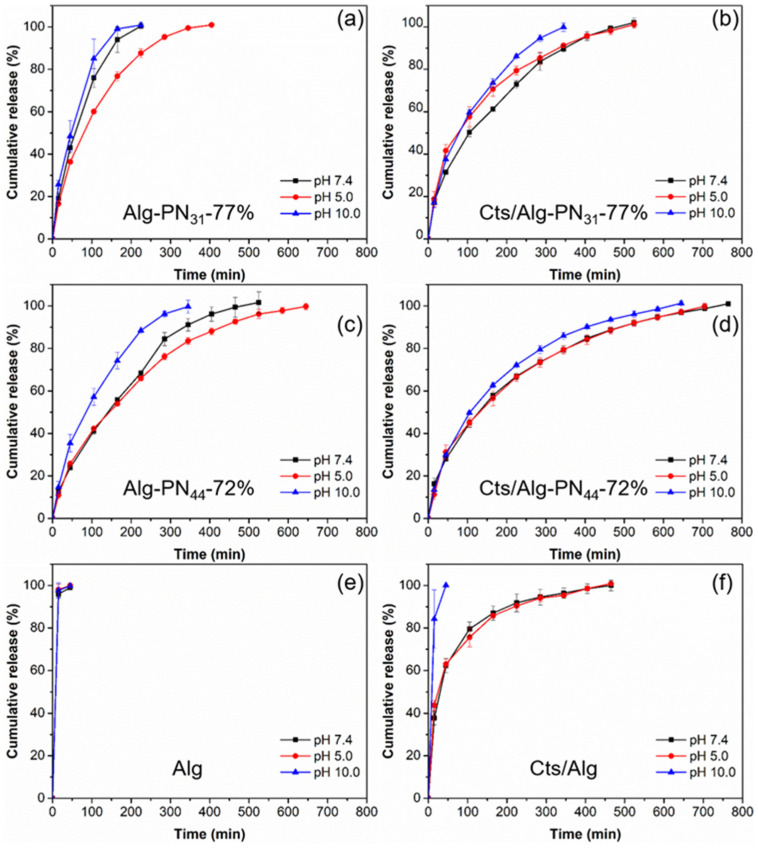
The release profiles of rhodamine B from the samples Alg-PN_31_-77% (**a**), Cts/Alg-PN_31_-77% (**b**), Alg-PN_44_-72% (**c**), Cts/Alg-PN_44_-72% (**d**), Alg (**e**), and Cts/Alg (**f**) at different pH and 37 °C.

Alginate and chitosan are pH sensitive polymers. The effect of pH on the release of RB from the PEC and non-PEC hydrogels were investigated using acetic buffer (pH 5.0) and borate buffer (pH 10.0). The comparisons of the release profiles for the hydrogels at different pH are shown in Figure 6. The release profiles for each hydrogel at different pH are shown in Figure 7.

Among the non-PEC hydrogels formed by copolymers, the hydrogel Alg-PN_31_-77% always gave the fastest release rate due to the short PNIPAAm chains as shown in Figure 5 and Figure 6. With the formation of PEC hydrogels, the release rate could be reduced. As a result, Cts/Alg-PN_31_-77% always gave slower release rate than Alg-PN_31_-77%, and same phenomena were observed for Cts/Alg-PN_44_-72% and Alg-PN_44_-72%. Moreover, the longer PNIPAAm chains also slowed down the release rate compared to shorter PNIPAAm chains. Therefore, Alg-PN_44_-72% gave a slower release than Alg-PN_31_-77%, and Cts/Alg-PN_44_-72% exhibited a slower release than Cts/Alg-PN_31_-77%. Interestingly, both PEC and non-PEC hydrogels formed from Alg-*g*-PNIPAAm with longer PNIPAAm chains showed less effects of pH changes on their release profiles. This is understandable because PNIPAAm is not a pH-sensitive polymer.

The acetic buffer (pH 5.0) could reduce the release rate for non-PEC hydrogels formed by copolymers alone, i.e., the Alg-PN_31_-77% and Alg-PN_44_-72% hydrogels (Figure 7a,c). Probably it is because that the acetic buffer could protonate the carboxylate group of alginate in the copolymers and reduce the hydrophilicity [37]. However, the acetic buffer increased the release rates for PEC hydrogels Cts/Alg-PN_31_-77% and Cts/Alg-PN_44_-72% (Figure 7b,d). In the PEC hydrogels, the number of deprotonated carboxyl groups of alginate and the number of pronated amine groups of chitosan are nearly balanced. The acetic buffer could destroy the balance by protonating the carboxylate groups of alginate. As a result, the PEC hydrogels became weaker and the release rate increased.

The borate buffer (pH 10.0) could neutralize the protonated amine groups of chitosan. Therefore, the high pH may weaken the PEC hydrogels and then increase the release rates for the PEC hydrogels. In the meantime, the borate buffer also deprotonate the non-PEC hydrogels formed by the Alg-*g*-PNIPAAm copolymers. Therefore, the high pH may also weaken the non-PEC hydrogels and then increase the release rate for the non-PEC hydrogels.

### 3.6. Release Kinetics and Mechanism

There are a number of mathematical models that are used to predict release kinetic models such as the zero order, first order, Higuchi, Korsmeyer–Peppas, and others [52]. In this study, it was found that the release kinetics data can fit well to the Krosmyer–Peppas model, which is also known as power law model describing drug release from a polymer matrix system with release mechanisms comprising diffusion of drug and/or water, swelling, and dissolution of the matrix. The Krosmyer–Peppas model is expressed by the following equation:

*M_t_*/*M*_0_ = *Kt^n^*

where *M_t_* is the total amount of RB released from the hydrogel at time *t*, *M*_0_ is the amount of RB loaded into the hydrogel, *M_t_*/*M*_0_ is the fraction of RB released from the hydrogel at time *t*, *K* is the release rate constant, and *n* is the release exponent. The value of *n* is related to the drug release mechanism [52].

For the case of a planar thin film system, *n* = 0.5 corresponds to a Fickian diffusion mechanism where the drug release is governed by diffusion, 0.5 < *n* < 1.0 to anomalous non-Fickian transport, *n* = 1.0 to non-Fickian Case II (relaxational) transport where the drug release rate corresponds to zero-order release kinetics and the mechanism driving the drug release is the swelling or relaxation of polymeric chains, and *n* > 1.0 to super case II transport mechanism [52].

The values of *n* and *K* were obtained by simulating the release profile data in Figure 7, which are listed together with the coefficient of determination (R^2^) in Table 4. The R^2^ values of the fittings are all higher than 0.979 while mostly higher than 0.99, indicating that the release kinetics data fitted well to the model. For all four samples with the fitting done in Table 4, the value of *n* ranges from 0.521 to 0.709. Taking the structures of the four hydrogel samples into consideration, the release should be governed by a combination of diffusion of RB and dissolution of the hydrogels. The PEC hydrogels Cts/Alg-PN_44_-72% and Cts/Alg-PN_31_-77% were stronger and stabler than their Alg-g-PN counterparts, so the release was mainly governed by diffusion with less dissolution. Particularly, at pH 7.4, the values of *n* for the PEC hydrogels Cts/Alg-PN_44_-72% and Cts/Alg-PN_31_-77% are 0.521 and 0.525, respectively, which are very close to *n* = 0.5. The *n* values are similar to those of the previously reported stable thermogel release system [58]. As discussed in previous section, at pH 5.0 and 10.0, the balance between the deprotonated carboxyl groups of alginate and the pronated amine groups of chitosan was impaired, and the hydrogels became weaker and dissociated faster than those at pH 7.4, therefore, the values of *n* for the PEC hydrogels are higher, ranging from 0.546 to 0.621, indicating the release mechanism contains more dissolution of the hydrogels. For the non-PEC hydrogels Alg-PN_44_-72% and Alg-PN_31_-77%, the values of *n* range from 0.613 to 0.709, indicating that the release was governed by more dissolution than those PEC hydrogel counterparts. The above discussion is in good agreement with the results on the weight percentage of residual polymers after 100% release of RB in (Table 3), where the hydrogels formed by Alg-g-PN alone eroded completely after the complete release of RB, while 40—70% of the PEC hydrogels remained after the complete release of RB.

## 4. Conclusions

Our data have demonstrated that the smart hydrogels were successfully prepared through formation of PEC between the negatively charged Alg-*g*-PNIPAAm copolymers and the positively charged chitosan in aqueous solutions. It was further proved that the PEC hydrogels were able to respond to both temperature and pH changes due to the natures of Alg-*g*-PNIPAAm and chitosan. Although the Alg-*g*-PNIPAAm copolymers could form very weak hydrogels by themselves, much stronger PEC hydrogels could be formed between the Alg-*g*-PNIPAAm copolymers and chitosan. The rheological study revealed that the strength of the PEC hydrogels was significantly enhanced through the formation of the PECs between the negatively charged alginate segments and the positively charged chitosan polymer chains. In addition, the existence of the PNIPAAm segments in the Alg-*g*-PNIPAAm copolymers significantly enhanced the strength of the PEC hydrogels upon temperature increasing over the LCST of PNIPAAm, because PNIPAAm would change from hydrophilic to hydrophobic upon increasing temperature over its LCST, and the hydrophobic interaction between the PNIPAAm segments may play a role as additional physical crosslinking. The length of the PNIPAAm in the Alg-*g*-PNIPAAm copolymers must be long enough to enhance the PEC hydrogels. The PEC hydrogels could sustain the release of the model drug RB from the hydrogels. Low pH may strengthen the non-PEC hydrogels formed by the Alg-g-PNIPAAm copolymers, and then result in more sustained release of RB from the hydrogels. However, low pH may weaken the PEC hydrogels, and resulted in less sustained release profiles. In contrast, high pH may weaken both PEC and non-PEC hydrogels formed by the Alg-*g*-PNIPAAm copolymers, and then result in less sustained release for the hydrogels. The PEC hydrogels showed more sustained release profiles than the corresponding non-PEC hydrogels. Each hydrogel showed the most sustained release at pH 7.4. Among all hydrogels tested in this study, the PEC Alg-PN_44_-72% hydrogel showed the most sustained release profiles. The release kinetics data were found to fit well to the Krosmyer–Peppas power law model. The analysis of the release kinetic parameters obtained by the modelling indicates that the release of RB from the PEC hydrogels followed mechanisms combining diffusion and dissolution of the hydrogels, but the release was mainly governed by diffusion with less dissolution at pH 7.4 when the PEC hydrogels were stronger and stabler than those at pH 5.0 and 10.0, as well as those non-PEC counterparts. Therefore, the PEC hydrogels formed by the Alg-*g*-PNIPAAm copolymers and chitosan are a kind of smart hydrogels that could respond to both temperature and pH changes, holding great potential for drug delivery applications.

## Figures and Tables

**Table 1:** List of the alginate-*g*-PNIPAAm copolymers and their molecular characteristics.

Sample ^1^	Mn of PNIPAAm ^2^	PDI of PNIPAAm ^3^	DS_NMR_ ^4^	PNIPAAm Content (wt%)
Alg-PN_31_-77%	3471	1.16	19.7	77
Alg-PN_44_-72%	4970	1.15	10.3	72

^1^ The copolymers are denoted Alg-PN_x_-y%, where Alg and PN represent alginate and PNIPAAm, respectively, x is the degree of polymerization and y% is the weight percentage of PNIPAAm in the copolymer. ^2^ The Mn of PNIPAAm was measured by GPC with THF as eluent. ^3^ “PDI” is the polydispersity index M_w_/M_n_. ^4^ “DS” is the degree of substitution, which is the number of the grafted PNIPAAm chains per 100 saccharide units in alginate. The DS was estimated by ^1^H NMR.

**Table 2:** Formulations of polymer samples use for PEC hydrogel formation.

Sample Name	Sample Type	Alginate (wt%)	PNIPAAm (wt%)	Chitosan (wt%)	Total Polymer (wt%)
Alg-PN_31_-77%	Copolymer hydrogel	1.7	5.7	0	7.4
Cts/Alg-PN_31_-77%	PEC hydrogel	1.4	4.8	1.2	7.4
Alg-PN_44_-72%	Copolymer hydrogel	2.1	5.3	0	7.4
Cts/Alg-PN_44_-72%	PEC hydrogel	1.7	4.3	1.4	7.4
Alg	Alg sol	1.7	0	0	1.7
Cts/Alg	PEC sol	1.7	0	1.4	3.1

**Table 3:** The weight percentage of residual polymers after 100% release of RB in PBS buffer (pH 7.4), acetic buffer (pH 5.0), and borate buffer (pH 10.0).

Sample	Weight Percentage of Residual Polymers (%)		
	PBS (pH 7.4)	Acetic Buffer (pH 5.0)	Borate Buffer (pH 10.0)
Alg-PN_31_-77%	0	0	0
Cts/Alg-PN_31_-77%	39.6 ± 4.8	23.9 ± 7.1	32.5 ± 4.0
Alg-PN_44_-72%	0	3.3 ± 1.1	0
Cts/Alg-PN_44_-72%	68.7 ± 19.9	37.1 ± 9.5	41.1 ± 0.6
Alg	0	0	0
Cts/Alg	49.3 ± 8.1	21.8 ± 4.0	33.1 ± 1.4

**Table 4:** Release kinetic characteristics of deferent hydrogel samples in PBS buffer (pH 7.4), acetic buffer (pH 5.0), and borate buffer (pH 10.0) ^a^.

Sample	PBS (pH 7.4)			Acetic Buffer (pH 5.0)			Borate Buffer (pH 10.0)		
	*n*	*K* (min^−1^)	R^2^	*n*	*K* (min^−1^)	R^2^	*n*	*K* (min^−1^)	R^2^
Alg-PN_31_-77%	0.709	2.83	0.999	0.663	2.81	0.997	0.613	4.83	0.999
Cts/Alg-PN_31_-77%	0.525	4.27	0.998	0.546	4.59	0.979	0.612	3.38	0.991
Alg-PN_44_-72%	0.622	2.32	0.999	0.653	1.97	0.996	0.672	2.50	0.993
Cts/Alg-PN_44_-72%	0.521	3.94	0.998	0.616	2.44	0.981	0.621	2.64	0.992
Alg	-	-	-	-	-	-	-	-	-
Cts/Alg	-	-	-	-	-	-	-	-	-

^a^ The release kinetics data are fitted to the equation *M_t_*/*M*_0_ = *Kt^n^* with *M_t_*/*M*_0_ < 0.7, where *M_t_* is the total amount of RB released from the hydrogel at time *t*, *M*_0_ is the amount of RB loaded into the hydrogel, *K* is the release rate constant, and *n* is the release exponent. R^2^ is the coefficient of determination for the data fittings. The samples Alg and Cts/Alg could not sustain the release of RB and the data are not suitable for the fitting.

## Data Availability

Data are available from the authors.

## Supplementary Materials

The following are available online at https://www.mdpi.com/article/10.3390/gels8070441/s1, Experimental section for materials, copolymer synthesis and general characterization methods. Figure S1: ^1^H NMR spectra of alginate, PNIPAAm, and alginate-*g*-PNIPAAm.

## Sample Availability

Samples of the compounds are available from the authors.
