# Supplementary material for: Smart Hydrogel Formed by Alginate-g-Poly(N-isopropylacrylamide) and Chitosan through Polyelectrolyte Complexation and Its Controlled Release Properties
Source: Gels. 2022 Jul 14;8(7):441. doi: 10.3390/gels8070441 (PMC9315676; doi:10.3390/gels8070441)
Supplement: Supplementary file 1 [file gels-08-00441-s001.zip › gels-1777891-supplementary.pdf]

gels

Supporting Information

Smart Hydrogel Formed by Alginate-g-Poly (*N*-Isopropylacrylamide) and Chitosan through Polyelectrolyte Complexation and Its Controlled Release Properties

Min Liu [1,2], Jingling Zhu [1], Xia Song [1], Yuting Wen [1] and Jun Li [1,2,*]

1. Experimental Section

Materials

N-Isopropylacrylamide (NIPAAm, TCI) was purified by recrystallization from hexane and dried at 100 °C under vacuum before use. Copper(I) bromide (CuBr), α-bromoisobutyric acid (BIBA), tris[2-(dimethylamino)ethyl]amine (Me_6TREN), sodium alginate (M/G = 1.56, Mn = 120k-190k Da), 2-(*N*-morpholino)-ethanesulfonic acid (MES), 2,4,6-trinitrobenzene sulfonic acid (TNBS), glycine, chitosan (Mn = 50k-190k Da), rhodamine B, and deuterium oxide (D_2O) were purchased from Sigma-Aldrich. 1-Ethyl-3-(dimethylaminopropyl)carbodiimide (EDC) and *N*-hydroxysuccinimide (NHS) were purchased from Tokyo Chemical Industry Co. Ltd. Diethyl ether, isopropanol, tetrahydrofuran (THF), methanol, and dichloromethane (DCM) were purchased from QReC.

Synthesis of Amino-Poly(N-isopropylacrylamide)

Polymerization of NIPAAm was conducted in 2-PrOH/water (3:1, v/v) mixed solvent. With target degree of polymerization (DP) of 38 and 50, the molar ratio of NIPAAm:BIBA:CuBr:Me_6TREN was = 38:1:1:1 and 50:1:1:1, respectively. NIPAAm, BIBA, Me_6TREN were dissolved into the mixed solvent and purged with nitrogen for 1 hour in ice bath. Then, CuBr was added quickly and purged for another half an hour. Then, the reaction continued in ice bath under nitrogen atmosphere for 1 hour before transferring the reaction tube into the 4 °C fridge. When the tube was opened, and catalyst was exposed to air, the reaction was stopped. The final green mixture obtained was diluted with THF and passed through a short Al_2O_3 column to remove copper catalyst. The resulting eluate solution was concentrated by rotary evaporator and then precipitated in diethyl ether. The white product was collected by centrifugation, and washed with diethyl ether. The product was dried under vacuum overnight.

The collected PNIPAAm was dissolved in DCM with EDC and NHS for 1 hour in ice bath. Then, the solution was added into ethylenediamine (EDA) in DCM solution dropwise in ice bath. The amount of EDC and NHS was determined by the molar ratio of PNIPAAm:EDA:EDC:NHS = 1:10:1:1. The final concentration of NHS was 0.05 mmol/mL. The final product was purified by precipitation in diethyl ether three times and dialysis in dialysis tube (molecular weight cutoff of 2000) for 3 hours and the solution was lyophilized.

Quantification of Free Amines in Amino-PNIPAAm by TNBS Assay

Sodium bicarbonate (0.1 M, pH 8.5) was prepared as reaction buffer. TNBS (0.01%, w/v%) was prepared using the reaction buffer as a diluent. SDS solution (10%, w/v%) was prepared in DI water. The amino-PNIPAAm was dissolved in the reaction buffer at a concentration of 1.2 mg/mL. About 0.25 mL of the TNBS solution was added to 0.5 mL of the amino-PNIPAAm solution. The mixture was incubated at 37 °C for two hours. Then, 0.25 mL of SDS solution and 0.125 mL of 1 N HCl were added to the mixture. The absorbance of the final solution was measured at 335 nm. Glycine (7.5 mg) was dissolved in 10 mL reaction buffer. Then, a series of known concentrations of glycine solutions was prepared

with 200 μM glycine solution and sodium bicarbonate buffer. The series of glycine solutions were then used to obtain a standard curve for the quantitative determination of free amines in amino-PNIPAAm.

Synthesis of Alginate-g-PNIPAAm

The dried amine-ended PNIPAAm was dissolved in MES buffer (10 mg/mL, pH 6.5). The alginate amount was determined by DP and degree of substitution (DS) value. The molar ratio of carboxyl group of alginate:EDC:NHS was = 1:1:1. The final product was purified by precipitation in methanol followed by dialysis in dialysis tube (molecular weight cutoff of 12000 Da) for 4 days. After dialysis, the solution was lyophilized.

Molecular Characterization

^{1}H NMR spectra were recorded on a Bruker AV-400 NMR spectrometer at room temperature. Chemical shift at 4.7 ppm was referred to the solvent peak of D_2O. Gel permeation chromatography (GPC) measurement was performed with a Shimadzu SIL-10A and LC-20AD system equipped with two Phenogel 10 μm 100 and 10000 Å columns (size: 300 × 7.8 mm) connected in series and a Shimadzu RID-10A refractive index detector. Tetrahydrofuran (THF) was used as the mobile phase at a flow rate of 0.6 mL/min at 40 °C. The system was calibrated using monodispersed PEG standards.

2.1. H NMR Analysis of Alg-g-PNIPAAm Copolymers

The characteristic peaks of PNIPAAm are located at 3.82 ppm (-NH-CH), 1.07 ppm (-CH_3), 1.94 ppm (-CH-), and 1.50 ppm (-CH_2-), while those of NIPAAm monomer between 5.0 and 6.0 disappeared. The conjugation between PNIPAAm-NH_2 and alginate was confirmed by ^{1}H NMR as shown in Figure S1. Three protons of the alginate ring (i.e., H2, H3, and H4) are overlapped with characteristic signals of PNIPAAm-NH_2 at around 3.8 ppm.

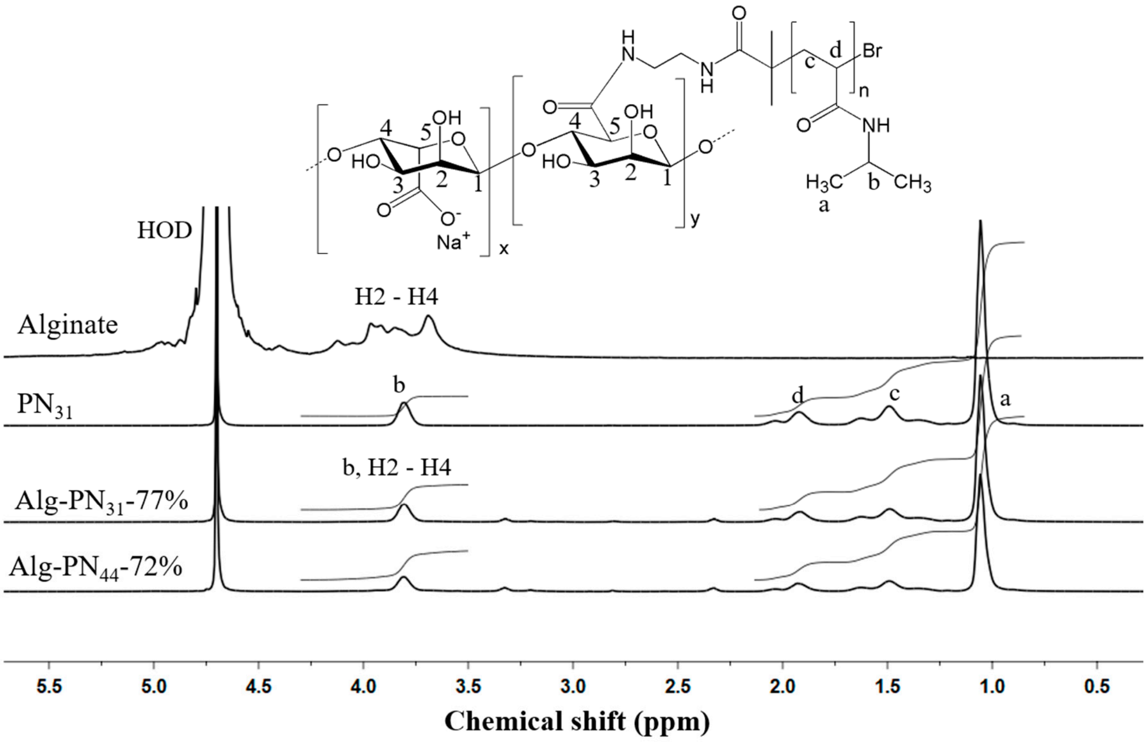

Figure S1. ^{1}H NMR spectra of alginate, PNIPAAm, and alginate-*g*-PNIPAAm in D_2O.
